# Outcomes of Urinary Tract Endometriosis—Laparoscopic Treatment: A 10-Year Retrospective Study

**DOI:** 10.3390/jcm12226996

**Published:** 2023-11-09

**Authors:** Maria Alexandra Rocha, Gonçalo Mendes, Luis Ferreira Castro, Sofia Mesquita, Bernardo Lobão Teixeira, Mariana Madanelo, João Aragão Vital, Miguel Marques-Monteiro, Nuno Vinagre, Beatriz Oliveira, Martinha Magalhães, Paulo Príncipe, Hélder Ferreira, Miguel Silva-Ramos

**Affiliations:** 1 Urology Department, Centro Hospitalar e Universitário de Santo António, 4099-001 Porto, Portugal; u12667@chporto.min-saude.pt (G.M.); sofiamesquita.urologia@chporto.min-saude.pt (S.M.); bernardoteixeira.urologia@chporto.min-saude.pt (B.L.T.); u13015@chporto.min-saude.pt (M.M.); u14790@chporto.min-saude.pt (J.A.V.); u13504@chporto.min-saude.pt (M.M.-M.); u14536@chporto.min-saude.pt (N.V.); u14879@chporto.min-saude.pt (B.O.); pauloprincipe.urologia@chporto.min-saude.pt (P.P.); u06537@chporto.min-saude.pt (M.S.-R.); 2 Gynecology Department, Centro Hospitalar e Universitário de Santo António, 4099-001 Porto, Portugal; u12611@chporto.min-saude.pt (L.F.C.); helder.ferreira@chporto.min-saude.pt (H.F.)

**Keywords:** endometriosis, urinary tract endometriosis, laparoscopy, ureter, bladder

## Abstract

Introduction: Urinary tract endometriosis (UTE), a rare manifestation, encompasses bladder and ureteral involvement. Surgical intervention is commonly recommended for UTE, though the optimal surgical approach remains a subject of debate. This study aims to report our centre’s experience with UTE. Methods: We conducted a retrospective cohort study of 55 patients who underwent surgical treatment for UTE at a single tertiary centre over a 10-year period (2012–2022). Patient data, including demographics, symptoms, intraoperative findings, and complications, were collected from medical records. Data were statistically analysed, and correlations were explored. Results: The study population had a mean age of 37.11 years, with dysmenorrhea (89.1%) being the most common symptom. Bladder endometriosis was present in 27 cases, ureteral endometriosis in 25, and mixed-location in 3. Laparoscopy was the primary surgical approach, with multidisciplinary teams involving urologists. There were six patients with postoperative complications, and there were six (10.9%) recurrences of endometriosis. A positive correlation was found between age and recurrence, but no significant predictors of recurrence were identified in our analysis. Conclusions: Laparoscopic treatment of urinary endometriosis is safe and effective. Multidisciplinary collaboration plays a pivotal role in addressing this challenging condition.

## 1. Introduction

Endometriosis is a gynaecological condition frequently observed in women of re-productive age. This disease is defined by the presence of endometrial glands and stroma outside of the uterine cavity, which undergo cyclic proliferation and breakdown as normal endometrial cells do [1,2]. The ensuing bleeding results in local inflammatory reaction, which leads to the formation of scar tissue and adhesions [2].

Endometriosis is considered a chronic disease and has considerable effects on patients’ quality on of life [3]. The main symptoms associated with endometriosis are dysmenorrhea, dyspareunia, and pelvic pain [2,4]. Despite the significant burden caused by this disease, the aetiology and pathogenesis of endometriosis are not completely understood [2,5]. The theories of endometriotic cells’ origin are the presence of retrograde menstruation, coelomic metaplasia, the spread of endometrium-derived stem/progenitor cells and altered genetic/epigenetic or immune factors [2,4,6].

Pelvic endometriosis is classified into three different phenotypes: ovarian endometrioma, superficial peritoneal lesions, and deep infiltrating endometriosis [3,4]. The latter is defined as the implantation of endometrial tissue penetrating the walls of any pelvic organ and/or retroperitoneal space extending >5 mm, such as the uterosacral ligaments, vagina, bowel, and urinary tract [3,7]. Therefore, urinary tract endometriosis (UTE) is one the forms of deep infiltrating endometriosis [8]. The expression ‘urinary tract endometriosis (UTE)’ refers to endometriotic implants of the bladder, ureter, kidney, and urethra [4]. The urinary tract is a rare location for endometriosis, and this type occurs in between 1.2 and 6% of cases [1,3], with bladder endometriosis corresponding to ~84% and ureter endometriosis to 15% [9].

Medical treatment in endometriosis, based on hormonal therapy, is more suppressive rather than curative [3,9]. The treatment of urinary tract endometriosis is generally accepted to be surgical, although no consensus has been achieved regarding which surgery to choose according to the location [3,9]. Ureter endometriosis can be managed with different types of surgeries, ranging from ureterolysis to reimplantation. With bladder endometriosis, the problem remains: it can be managed with partial cystectomy, transurethral resection, or excision of the nodule [3,4,7,8,9,10,11,12]. Due to the scarcity of cases and studies, the choice of surgical management remains a challenge. [11]

The main objective of our study is to report our centre’s experience with urinary tract endometriosis, including our surgical management, occurrence of postoperative complications, and recurrence rate.

## 2. Methods

We conducted a retrospective cohort study of patients who underwent surgery for urinary tract endometriosis (UTE) at a single tertiary centre between 2012 and 2022.

All clinical and surgical data were collected from patients’ files, focusing on preoperative state, namely patients’ age, previous surgeries, hormonal therapy, the presence of auto-immune disease, and the presence of symptoms. All patients had UTE confirmed on the pathology report. The endometriosis was classified according to its location in the bladder, ureter, or mixed locations (in cases where both the bladder and ureter were affected). All cases with UTE were managed based on their specific location and intraoperative findings. A laparoscopic approach was used initially in all cases. In the cases of ureteral endometriosis, ureterolysis was used for extrinsic compression with endometrial nodules, and in cases where a stricture was observed, ureteral end-to-end anastomosis or ureteral reimplantation was performed. No aiding method to detect or highlight the ureters, like intraureteral indocyanine green, was used in any case. In cases of bladder endometriosis, the surgeons performed partial cystectomy, excision of the nodule (without opening the bladder), or transurethral resection of the nodule, according to the infiltrating grade of the lesion. These data can be seen in Table 1.

Other data collected included the presence of a urology specialist in the surgical team, the intraoperative time, and the occurrence of complications during the surgery. The postoperative complications were recorded, including the first 30 days, and classified according to the Clavien–Dindo classification.

Endometriosis recurrence was defined as the reappearance of an endometrioma on imaging (US or MRI) and was assessed by consulting the clinical file.

Data were collected in an SPSS file. We calculated descriptive statistics for all the variables. The continuous variables’ central measures are presented as a mean or median, according to the presence of a normal distribution. The categorical and nominal variables are presented in frequency tables. The correlation between variables was ascertained using the Pearson correlation and univariable analysis. Differences were considered statistically significant if *p* < 0.05.

## 3. Results

We retrospectively collected data from 55 women who underwent surgery for UTE between 2012 and 2022 in our tertiary centre.

The patients had a mean age of 37.11 ± 5.44 years, a mean weight of 62.11 (±11.47) kg, a mean height of 1.63 (±0.06) m, and a mean BMI of 23.29 ± 4.00. Regarding the fertility of the women analysed, we encountered a mean of 0.55 (median 0 (0–4)) gestations, with a mean number of labours of 0.34 (median 0 (0–2)) and an abortion number of 0.19 (median of 0 (0–2)).

The most-reported symptom was dysmenorrhea (89.1%), followed by dyspareunia (58.2%), dysuria (40%), and pelvic pain (25.5%). Only one patient reported lumbar pain (1.8%). Sorting the patients by endometriosis location, in bladder endometriosis, we had 25 patients with dysmenorrhea, 17 patients with dysuria, 14 with dyspareunia, and 4 patients with pelvic pain. In the ureteral endometriosis, we found 21 patients with dysmenorrhea, 16 with dyspareunia, 9 with pelvic pain, 3 with dysuria, and 1 with lumbar pain. In mixed-location endometriosis, three had dysmenorrhea, two had dyspareunia and dysuria, and one had pelvic pain. This information can be seen in Table 2.

Of the 55 patients, 28 had a history of previous surgery (see Table 3 for details). Regarding the previous therapy, 34 (61.8%) took oral progestogens (oral hormonal contraceptives), 1 took triptorelin (1.8%), and 20 (36.4%) had no previous medication for endometriosis. A history of endometriosis was present in 20 women (36.4%), and 5 had the diagnosis of an autoimmune disease (9.1%).

With regard to the location, we had 27 cases of bladder endometriosis, 25 of ureteral endometriosis, and 3 with a mixed location. Considering the size of all the urinary tract endometriosis, we had a mean size of 25.58 (±17.17) mm.

Bladder endometriosis was further classified according to the location in the bladder: 23 cases in the posterior wall, 6 cases in the dome, and 1 case in the right lateral wall. The mean size was 24.60 (±11.11) mm.

Regarding the ureteral endometriosis, we had 23 cases located in the distal ureter, 4 cases in the middle ureter, and 1 case at the ureteral–vesical junction. The mean size was 26.18 (±21.51) mm. The ureteral cases were located on the left side in 16 cases, on the right side in 11 cases, and we had 1 case of bilateral endometriosis. In 19 of the cases, hydronephrosis was detected.

All surgeries were performed using the laparoscopic approach, with only one case of conversion to open procedure (laparotomy). According to the extension of the disease intraoperatively, in cases of bladder endometriosis, 14 patients underwent removal of the bladder endometrioma and 13 were submitted to a partial cystectomy. In ureteral endometriosis cases, the surgeons performed 15 ureterolysis, 7 end-to-end ureteral anastomosis, and 3 ureteral reimplantation procedures. In the mixed-location cases, a combined partial cystectomy and ureterolysis was the surgery of choice twice, and ureterolysis with transurethral resection of the endometriosis nodule was chosen for the other case. The laparoscopy conversion to laparotomy was a case of ureteral endometriosis with associated bowel endometriosis, and the surgeries performed were an end-to-end ureteral anastomosis and an anterior rectal resection with protection ileostomy.

In most of the cases, the endometriosis was not limited to the urinary tract, and so other surgeries were performed in 47 cases. The most frequent surgery was removal of an endometriosis nodule from the recto-vaginal septum, in 25 (45.5%) patients, followed by removal of an endometriosis nodule from the parametrium and uterosacral ligaments in 13 (23.6%). These data, and the other surgeries performed, can be seen in Table 4. There was isolated urinary tract endometriosis in eight of the described cases.

The presence of the urology surgical team in the theatre was also recorded: in 41 cases, the urologist was part of the surgical team from the beginning, in 2 cases, they were called in an urgent manner, and in 12 cases, there was no record of a urologist being in the operating theatre. We found no correlation between the occurrence of intraoperative or postoperative complications and the presence of the urology team (*p* = 0.273 and *p* = 0.746, respectively).

The mean operative time was 149.09 (±76.88) min. We found no correlation between the operative time and the presence of bladder endometriosis or ureteral endometriosis (*p* = 0.35 and *p* = 0.28, respectively).

There was an intraoperative surgical incident in four cases, three with an iatrogenic lesion of the bladder and one with a lesion of the ureter. All cases of bladder lesions were in patients with ureteral endometriosis, and the case of ureteral lesion happened in a mixed-location endometriosis case. We found no correlation between the size of the lesion and its location and the occurrence of intraoperative lesions (*p* = 0.26 and *p* = 0.75, respectively).

There were six cases of complications postoperatively in the first 30 days. The clinical findings and subsequent treatment can be found in Table 5. We found no correlation between the occurrence of postoperative complications and the location of endometriosis (*p* = 0.14) or the surgery for urinary tract endometriosis (*p* = 0.42).

During follow-up, we found that only 12 patients (21.8%) were on oral progestogens. The recurrence rate was 10.9%, with six patients affected. The recurrence was found in the ovary in three cases, in the recto-vaginal septum in two, and there was one bowel recurrence. No cases of UTE were found in recurrence. The time to recurrence was 72 (24; 92) months. We found a positive linear correlation between age and the occurrence of recurrence (*p* = 0.04). We were not able to find a correlation between the recurrence of endometriosis and BMI (*p* = 0.18), the use of progestogens prior to the surgery (*p* = 0.42), the presence of autoimmune disease (*p* = 0.41, OR: 1.14), the presence of previous endometriosis (*p* = 0.42), the location of endometriosis (*p* = 0.54), the size of the lesion (*p* = 0.96), or the use of postoperative progestogens (*p* = 0.73). When combining all these variables in a logistic regression, none of the predictor variables are statistically significant in predicting endometriosis recurrence. This suggests that the data do not provide sufficient evidence to conclude that any of the tested variables have a significant effect on the likelihood of recurrence.

## 4. Discussion

This study reports the results of a retrospective cohort study on 55 patients with urinary tract endometriosis submitted to surgery on our tertiary centre between 2012 and 2022.

Our patients had a mean age of 37.11 ± 5.44 years, which is included in the reproductive years [7,9]. In terms of fertility, we found a median gestation number of zero. As described in the literature, there is an increased incidence of infertility in patients with endometriosis [4,6,13]. Our results are in line with the described, highlighting the importance of early diagnosis and treatment. Despite the importance of fertility issues in patients with endometriosis, our study did not focus on this problem.

Endometriosis is a disease with no specific symptoms [12]. Bladder endometriosis is usually associated with urinary symptoms such as dysuria or urinary frequency [8]. In endometriosis involving the ureter, usually, there are no specific symptoms [8]. In our series, the most frequent symptom was dysmenorrhea, regardless of the location of the endometriosis, followed by dyspareunia. These symptoms are not specific to urinary tract endometriosis, or even endometriosis, which highlights the fact that this disease has no pathognomonic signs [12] and explains some delay in the diagnosis. We had 17 patients with bladder endometriosis complaining of dysuria, a sign linked to this condition. Other storage symptoms were not recorded evenly throughout the clinical records, and so could not be accessed in this work. Regarding ureteral endometriosis, it is usually asymptomatic and can lead to kidney failure [8,12]. In our cases of ureteral endometriosis, most of the patients had dyspareunia and dysmenorrhea, likely due to endometriosis located outside the urinary tract.

In 36.4% of the cases, there was previous history of endometriosis, which means 36.4% of our cases are recurrences. It is important to note that the precise definition of endometriosis recurrence lacks a standardized consensus and has been a subject of discussion and research over recent years. As a result, the reported recurrence rate varies considerably [10,14,15]. This variability is attributed to the diverse definitions of recurrence used across studies and the nature of the research methodologies used. Our data did not prove there was a correlation between the history of previous endometriosis and the recurrence of this disease (*p* = 0.42), as stated previously.

In our patients, five had a diagnosis of an autoimmune disease (9.1%). It has been hypothesized that the pathogenesis of endometriosis is linked to an immune-mediated process [13,16], as women with this disease demonstrate altered immune surveillance [5]. Considering this hypothesis, we tested the relationship between the occurrence of recurrence and the presence of autoimmune disease in our cohort and found no statistically significant correlation (*p* = 0.41). This difference may be due to the small number of patients with autoimmune diseases in our cohort.

Urinary tract endometriosis is most frequently reported in the bladder (~84%), followed by ureteral endometriosis in about 15% of cases [4,9,17]. On the contrary, we found a similar distribution between the cases of bladder and ureteral endometriosis, with 27 cases of the first and 25 cases of the latter (and 3 with mixed location). This difference may be mainly attributed to the small group of patients and to the fact that only surgically treated cases of urinary tract endometriosis were included.

In our series, all cases were managed through laparoscopy (with only one conversion to laparotomy). The European Society of Human Reproduction and Embryology guidelines on endometriosis state that surgical treatment is an important part of the management of endometriosis [12]. Historically, open surgery was the first choice, but in recent decades, laparoscopy has dominated [12,18]. Nevertheless, the best surgical treatment is yet to be defined. Nehzat et al. suggest that the optimal approach for urinary tract endometriosis is laparoscopic, either with or without robot assistance [1]. In our centre, and due to the extensive experience in laparoscopic surgery, we agree with this statement (although there is no experience in robotic assistance to date). The management of this disease requires a team experienced in laparoscopy, and this team should be composed of gynaecologists and urologists. According to our records, a urologist was present in the operating theatre in ~78% of cases, including two cases requiring their urgent presence. We advocate the need for a multidisciplinary team in the surgical room to manage these complex surgeries and patients.

We had six (10.9%) cases of postoperative complications. According to the Clavien–Dindo classification, two patients had grade I and two had grade II complications. The other two cases, classified as grade IIIb, required additional surgery to address the complication. Overall, we believe this to be a low complication rate, despite the limited number of patients in our study. We were not able to find a correlation between the existence of complications and the location of the urinary tract endometriosis. This observation is in line with the results of the FRIENDS group [18].

During the follow up of our patients, we found that only 12 patients were on oral progestogens, namely continuous oral contraceptives. It is recommended, after surgical excision of endometriomas, in women not seeking conception, to offer long-term hormone treatment, like hormonal contraceptives, for the secondary prevention of endometrioma and endometriosis-associated related symptom recurrence [18]. These data should be regarded as an opportunity to improve our care in patients with endometriosis, as our rate of postoperative hormonal therapy is low. Patients should be informed about the potential for recurrence, particularly if they do not to pursue hormonal suppression following surgery [1].

Our recurrence rate was 10.9%. In the literature, the recurrence rate varies widely, as stated before. Saavalainen et al. reported that only ~4% of recurrences were of UTE [8]. In our series, no cases of recurrence were found in the urinary tract.

In our data, we found a positive correlation between age and recurrence, but could not find any statistical significance for predictors of recurrence. Intriguingly, a study conducted by Di Maida et al. yielded contrasting results, indicating that younger age could serve as a predictor for recurrence [3]. It is important to note that this disparity in findings might be attributed to the relatively small sample size in our study, underscoring the need for further research with larger cohorts to provide more conclusive insights into the relationship between age and endometrioma recurrence.

## 5. Conclusions

In conclusion, this retrospective cohort study provides insight on the intricate landscape of urinary tract endometriosis (UTE) management and outcomes within our centre over a period of 10 years. Our comprehensive analysis of 55 cases underscores the efficacy of laparoscopic interventions and the need for a multidisciplinary team to manage these patients. Recurrence rates remain variable, with age potentially playing a role, though further research is needed to clarify predictive factors.

Our findings provide valuable insights into the evolving knowledge in this field, emphasizing the need for continued research and collaboration in the quest for improved UTE management strategies. In the future, we aim to add robotic-assisted management and to increase our amount of data.

## Figures and Tables

**Table 1:** Surgeries performed according to the classification of endometriosis.

Classification of Endometriosis	Surgery Performed
**Ureteral endometriosis**	Ureterolysis
	Ureteral end-to-end anastomosis
	Ureteral reimplantation
**Bladder endometriosis**	Partial cystectomy
	Excision of the nodule
	Transurethral resection of the nodule

**Table 2:** Symptoms presented by the patients with UTE.

Symptoms	Frequency (Percentage)
**Dysmenorrhea**	**49 (89** **.** **1%)**
Bladder endometriosis	25
Ureteral endometriosis	21
Mixed-urinary-location endometriosis	3
**Dyspareunia**	**32 (58** **.** **2%)**
Bladder endometriosis	14
Ureteral endometriosis	16
Mixed-urinary-location endometriosis	2
**Dysuria**	**22 (40%)**
Bladder endometriosis	17
Ureteral endometriosis	3
Mixed-urinary-location endometriosis	2
**Pelvic pain**	**14 (25** **.** **5%)**
Bladder endometriosis	4
Ureteral endometriosis	9
Mixed-urinary-location endometriosis	1
**Lumbar pain**	**1 (1** **.** **8%)**
Bladder endometriosis	0
Ureteral endometriosis	1
Mixed-urinary-location endometriosis	0

**Table 3:** Data collected from our patients regarding previous surgeries.

Previous Surgery		Frequency (Percentage)
**Gynecological surgeries**	Diagnostic laparoscopy	5 (9.1%)
	Hysterectomy with bilateral oophorectomy	3 (5.5%)
	Salpingectomy with removal of ovarian cyst	2 (3.6%)
	Removal of ovarian endometriosis cyst	2 (3.6%)
	Laparoscopic oophorectomy	1 (1.8%)
	Conization of the cervix	1 (1.8%)
	Salpingectomy due to ectopic pregnancy	1 (1.8%)
	Removal of endometriosis nodule from uterosacral ligament and vesico-uterine peritoneum	1 (1.8%)
	Removal of endometriosis nodule from recto-vaginal septum and bladder	1 (1.8%)
**Urological surgeries**	Retrograde ureteral catheterization	4 (7.3%)
	Ureteral reimplantation	1 (1.8%)
	Partial cystectomy with removal of endometriosis cyst	1 (1.8%)
	Percutaneous nephrostomy tube	1 (1.8%)
	Transurethral resection of endometriosis nodule	1 (1.8%)
**Other surgeries**	Cholecystectomy	2 (3.6%)
	Removal of endometriosis nodule from abdominal wall with flap	1 (1.8%)

**Table 4:** Concomitant endometriosis surgeries performed.

Surgery	Frequency (Percentage)
Removal of endometriosis nodule from recto-vaginal septum	25 (45.5%)
Removal of endometriosis nodule from the parametrium and uterosacral ligaments	13 (23.6%)
Anterior rectal resection with protection ileostomy	2 (3.6%)
Removal of ovarian endometriosis cyst	2 (3.6%)
Hysterectomy with oophorectomy	1 (1.8%)
Hysterectomy	1 (1.8%)
Drainage of endometriosis cyst	1 (1.8%)
Salpingectomy and removal of endometriosis nodule	1 (1.8%)
Removal of endometriosis cyst from the diaphragm	1 (1.8%)

**Table 5:** Postoperative complications.

Symptoms/Findings	Location of Endometriosis	Treatment	Clavien–Dindo Classification
Lumbar pain and hydronephrosis ^1^	Ureter	Retrograde ureteral catheterization	IIIb
Peri-uterine hematoma ^2^	Ureter	Conservative approach and antibiotics	II
Anastomotic ureteral leakage ^3^	Ureter	Conservative approach	I
Haematuria ^4^	Ureter	Conservative approach (hydration)	I
Urinary (bladder) fistula ^5^	Bladder	Urethral catheterization	II
Anastomotic ureteral leakage ^6^	Ureter	Diagnostic laparoscopy with intra-abdominal drainage and percutaneous nephrostomy tube placement	IIIb

^1^ Case of ureter endometriosis. The urologic surgery was ureterolysis, and the concomitant surgery was hysterectomy with oophorectomy. ^2^ Case of ureter endometriosis. The urologic surgery was ureterolysis, and the concomitant surgery was removal of endometriosis nodule from the parametrium and uterosacral ligaments. ^3^ Case of ureter endometriosis. The urologic surgery was end-to-end ureteral anastomosis, and the concomitant surgery was anterior rectal resection with ileal protection (converted to laparostomy). ^4^ Case of ureter endometriosis. The urologic surgery was ureterolysis, and the concomitant surgery was drainage of endometriosis cyst. ^5^ Case of bladder endometriosis. The urologic surgery was removal of the bladder endometriosis nodule, and the concomitant surgery was removal of endometriosis nodule from the recto-vaginal septum. ^6^ Case of ureter endometriosis. The urologic surgery was end-to-end ureteral anastomosis, and the concomitant surgery was removal of endometriosis nodule from the parametrium.

## Data Availability

No new data were created or analyzed in this study. Data sharing is not applicable to this article.
